# Individualizing beta-lactam dosing in real-world patients: lessons from a pharmacist-led programme implementation and evaluation

**DOI:** 10.1093/jacamr/dlag047

**Published:** 2026-04-08

**Authors:** Samantha Pan, Erin Weslander, Sarah Fierek, Mark Ball, Christopher A Crutchfield, Shannon Galvin, Nathaniel J Rhodes

**Affiliations:** Department of Pharmacy, Northwestern Memorial Hospital, Chicago, IL, USA; Department of Pharmacy, Northwestern Memorial Hospital, Chicago, IL, USA; Department of Pharmacy, Northwestern Memorial Hospital, Chicago, IL, USA; Department of Pathology, Northwestern Memorial Hospital, Chicago, IL, USA; Department of Pathology, Northwestern Memorial Hospital, Chicago, IL, USA; Department of Pathology, Northwestern University Feinberg School of Medicine, Chicago, IL, USA; Division of Infectious Diseases, Department of Internal Medicine, Northwestern University, Chicago, IL, USA; Department of Pharmacy, Northwestern Memorial Hospital, Chicago, IL, USA; Department of Pharmacy Practice, Midwestern University, College of Pharmacy, Downers Grove, IL, USA; Pharmacometrics Center of Excellence, Midwestern University, Downers Grove, IL, USA

## Abstract

**Background:**

Individualized dosing of beta-lactam using serum concentrations is an emerging approach to overcome PK/PD variability in select patients. Herein, we describe a beta-lactam dose individualization programme highlighting successes and challenges.

**Methods:**

We conducted a single-centre evaluation of a beta-lactam dose individualization using serum concentration monitoring programme. In September 2023, a pharmacist-driven protocol was implemented at an academic centre as a collaboration between Infectious Diseases, Pharmacy, and the Clinical Laboratory. De-centralized pharmacists ordered serum concentrations for cefepime, meropenem or piperacillin-tazobactam treated patients.

**Results:**

Interpretation was conducted using Bayesian software. The drug monitored, treatment indication, model fitness, whether therapy was altered, whether protocols were followed and PK/PD attainment for each patient were recorded. Eighty-two beta-lactam serum levels were ordered in 47 patients between 26 September 2023 and 31 March 2024. Cefepime was most frequently monitored (70.7%). Common treatment indications in monitored patients were hospital-acquired/ventilator-associated pneumonia (25.5%), surgical prophylaxis (12.8%), bone/joint (12.8%) and central nervous system infection (12.8%). Model fitness was good or intermediate in >95% of cases. Dose or agent optimization was possible in 60% of cases, and dose adjustments were made in 34% of cases. Protocol deviations were common (57.4%). Targets of 100% *f*T_>1×MIC_ and *f*T_>4×MIC_ were achieved in 91.5% and 61.7% of patients, respectively. Troughs exceeding protocol safety goals occurred >20% of the time.

**Conclusion:**

We found that dose optimization was possible in >50% of monitored patients necessitating dose adjustments >33% of the time. Protocol deviations reinforced areas for continued pharmacist and provider education to optimize the use of this targeted intervention.

## Background

Beta-lactam antibiotics are a cornerstone of treatment for patients with serious infections. Management of infectious diseases involves a triad of the host, the infecting organism, and the antimicrobial chosen.^1^ Clinical pharmacokinetic and pharmacodynamic (PK/PD) studies have linked lower rates of PK/PD target attainment with an increased risk of worse outcomes for beta-lactam treated patients.^2–4^ Specifically, critically ill patients are at risk for pharmacokinetic changes that require adjustments to standard beta-lactam dosing regimens between 20% and 60% of the time depending on drug and renal disposition.^5^ Individualized beta-lactam dosing using therapeutic drug monitoring (TDM) has been described,^6^ but the use of this type of monitoring has lagged in the USA.^7^ In contrast to TDM, model-informed precision dosing (MIPD) builds upon principles of TDM while integrating past experience (e.g. population models using Bayesian priors) to improve predictions and facilitate dose adjustment.^8^ MIPD is an emerging approach to address variability in beta-lactam exposure that adversely affects PK/PD target attainment in patients; however, multiple challenges can emerge when implementing TDM or MIPD in real-world clinical practice.^9^

TDM is standard of care for antimicrobial drugs with widely recognized narrow therapeutic ranges such as vancomycin and the aminoglycosides. However, a clinical gestalt is that beta-lactams are safe and effective for many patients and thus do not require TDM. Barreto *et al.* found that individual internalization of TDM beliefs and organizational features (e.g. lack of support, lack of access to real-time assays) can present barriers to implementation of focused beta-lactam TDM or MIPD programmes in spite of conceptual enthusiasm for the clinical use of this tool.^9^ Whereas evidence supporting a benefit of beta-lactam TDM or MIPD have been inconsistent across clinical studies,^10^ toxicity with contemporary dosing approaches for beta-lactams has become increasingly apparent in practice.^11^ Patients manifesting beta-lactam neurotoxicity have longer lengths of stay and worse outcomes,^11^ underscoring the need for careful assessment of actual drug exposures in selected patients: that which can only be achieved using a patient-specific concentration evaluation.

In 2023, our academic medical centre developed, validated and implemented a real-time, in-house, beta-lactam assay for cefepime, meropenem and piperacillin. A protocol for the use of this laboratory developed assay was created as a partnership between clinical pharmacists, the Clinical Laboratory, and the Division of Infectious Diseases and was approved by the Pharmacy and Therapeutics subcommittee. We integrated the assay results into clinical workflows wherein clinical pharmacists identified patients in need of serum concentration monitoring and dose optimization using MIPD software. Herein, we characterize patient and process evaluation after implementation of a pharmacist-led beta-lactam dose individualization programme.

## Methods

### Design

We conducted a single-centre, retrospective, descriptive evaluation of patients who underwent beta-lactam dose individualization at a Northwestern Memorial Hospital (NMH), a 1000-bed academic medical centre. Patients admitted to NMH between 26 September 2023 and 31 March 2024 who were 18 years and older, treated with cefepime, meropenem or piperacillin-tazobactam at the discretion of their treatment team, and underwent protocolized pharmacist-driven PK sampling were included. Patients who were incarcerated or pregnant were excluded. Data elements were extracted from the electronic health record (Epic, Verona, WI, USA) or from the MIPD software program (InsightRX, San Francisco, CA, USA). The study was reviewed and approved by the Northwestern University IRB (protocol: STU00221348). Further de-identified data analysis was conducted at Midwestern University (determined to be non-human subjects research).

### Beta-lactam dose individualization protocol

The NMH Pharmacy and Therapeutics Committee approved a protocol for pharmacist-driven TDM for beta-lactam treated patients in July 2023. This workflow for beta-lactam TDM with in-house drug assay availability and MIPD support went live on 26 September 2023. De-centralized, unit-based pharmacists were trained on the protocol in September 2023 and provided education related to the populations of interest, the rationale for the recommended targets and the processes related to serum concentration ordering and interpretation. Pharmacists were responsible for evaluation of serum concentration data and documentation of dose adjustments according to protocol (available at adsp.nm.org). Dose adjustments were encouraged to be limited to standardized dosing regimens in alignment with institutional renal dosing guideline and extended infusion policies for ease of operationalizing orders in the EHR. Dose adjustments exceeding renal dosing guideline regimens or FDA labelled dosing were considered on a case-by-case basis in consultation with an infectious disease pharmacist. During this study period, there were no occurrences of extenuating dose recommendations. Pharmacists relayed all recommendations to the patient’s primary team and placed a progress note in the chart. An overview of the protocolized processes are shown in Supplementary Figure S1 (available as Supplementary data at *JAC-AMR* Online).

### Serum drug assay

Serum drug concentration analysis was performed in the Clinical Laboratory at NMH by means of a fully validated assay. Assays were validated in accordance to the FDA Bioanalytical Method Validation Guidance for Industry.^12^ Details on serum drug assay development can be found in the Supplement. Samples were run in daily batches on weekdays. Results were reported and populated within the patient’s medical record after each batch was completed and approved. Turnaround time ranged from 3 to 5 hours from the cut-off time for submission of samples to the laboratory of 9:00 AM.

### MIPD analysis

Serum concentrations for each drug were interpreted using an MIPD software (InsightRX) to estimate Bayesian posterior predicted PK profiles for each patient. Patient data including covariate values (e.g. CrCL, body weight), dose and the observed serum concentration data were entered into the software by clinical pharmacists to generate Bayesian posterior PK predictions. Subsequent dose adjustments were made by individual pharmacists using the Bayesian PK model predictions as a guide in conjunction with institutional protocols and suggested targets for safety and efficacy. Per the protocol, safety targets were defined for each drug based on trough concentrations (i.e. *C*_min_)^6,13^ and efficacy targets were based on the free time above 1×  and 4×  the MIC (*f*T_>1×MIC_ and *f*T_>4×MIC_)^6,13^ using published protein binding values to determine the free fraction. These PK/PD targets served as surrogate process endpoints (see the ‘Evaluation metrics’ section) in accordance with available literature.^10^ Safety targets defined in the protocol were extrapolated from suggested upper bounds for plasma concentrations, are institution specific and conservative. Of note, the suggested upper bounds have not been well-validated, and clinical judgement remains encouraged per protocol. When available, clinical microbiology laboratory defined MICs for infecting isolates were used (Vitek 2, Biomerieux, Marcy l'Étoile France). Otherwise, a worst-case scenario was assumed where the CLSI breakpoint MIC for *Pseudomonas aeruginosa* was used.^14^

### Data elements

For each PK sampling event, we characterized the following: ordering provider specialty, whether sampling was performed per protocol, whether PK sampling led to dosing changes, treatment indication, culture information, overall PK model fitness and estimated empirical PK/PD target attainment for efficacy and safety goals. Empiric dosing was guided by renal-function protocols and dose adjustments were made empirically using standard adult doses and intervals (Table S2). Multiple levels from the same patient that were ordered for one BL TDM event (e.g., peak and trough ordered) were categorized as one unique patient. Repeated BL TDM events were included as unique patients only if >7 days had passed in between events or if there was a significant change in clinical status or renal function. Protocol deviations were defined as sampling events that did not complete the following elements of the protocol: pharmacist-led ordering of BL TDM level, dose adjustment made per protocol, and progress note documenting recommendation published in a patient’s chart.

### Evaluation metrics

The rate of 100% *f*T_>1×MIC_ target attainment across measured beta-lactams during the first 24-hour period was the primary endpoint in this cohort. We also assessed the proportion of patients achieving 100% *f*T_>4×MIC_, the proportion of patients who received a dose adjustment (and the direction of adjustment), the proportion of patients who had a change in treatment agent(s), and the proportion of patients who received serum concentration monitoring according to our institutional protocols. The prevalence of trough concentrations exceeding protocolized safety trough targets (i.e. cefepime >20 mg/L, piperacillin >160 mg/L, or meropenem >20 mg/L) was also evaluated. Individual PK parameters and individual covariate relationships were assessed visually. The accuracy (median percentage error) and precision (median absolute percentage error) were assessed for CrCL versus drug clearance estimates.

### Data analysis

Descriptive statistics were calculated as means and standard deviations for continuous data or as counts and proportions for categorical data. Data were stored and analysed in Microsoft Excel (Redmond, WA, USA).

## Results

### Patients

Eighty-two beta-lactam serum concentrations were obtained in 47 patients during the study period. As shown in Table 1, the serum concentrations were most commonly obtained for cefepime (70.7%, *n* = 58/82), followed by meropenem (25.6%, *n* = 21/82), and rarely for piperacillin (3.7%, *n* = 3/82). Among included patients, the most common treatment indications were hospital-acquired/ventilator-associated pneumonia (25.5%, *n* = 12/47), surgical prophylaxis (12.8%, *n* = 6/47), bone/joint (12.8%, *n* = 6/47) and central nervous system (CNS) infection (12.8%, *n* = 6/47). Patients were frequently admitted to the ICU (31.9%, *n* = 15/47) or receiving care post-lung transplant (21.3%, *n* = 10/47). Only one patient had two unique BL TDM events included since these events were >1 month apart.

**Table 1.** Patient demographics and characteristics of infections

Patient characteristics	Frequency (*n*, %)
Indications, *n* = 47 patients	
HAP/VAP	12 (25.5)
Surgical prophylaxis	6 (12.8)
Bone/joint	6 (12.8)
CNS infection	6 (12.8)
Bacteraemia	4 (8.5)
SSTI	3 (6.4)
UTI	2 (4.3)
IE	2 (4.3)
Febrile neutropenia	2 (4.3)
CAP	2 (4.3)
Empyema	1 (2.1)
IAI	1 (2.1)
Primary service team, *n* = 47 patients	
ICU	15 (31.9)
Lung transplant	10 (21.3)
Surgery	8 (17)
HSCT	4 (8.5)
Internal medicine	4 (8.5)
Haematology/oncology	3 (6.4)
Abdominal transplant	3 (6.4)
Culture specimen type, *n* = 47 patients	
Respiratory BAL	11 (23.4)
Surgical culture	5 (10.6)
Blood	4 (8.5)
Donor culture	4 (8.5)
Wound culture	4 (8.5)
Sputum	3 (6.4)
CSF	2 (4.3)
Urine	2 (4.3)
Body fluid	2 (4.3)
Trach aspirate	1 (2.1)
No culture	9 (19.1)
Organism on culture, *n* = 47 patients	
*Pseudomonas aeruginosa*	13 (27.7)
*Escherichia coli*	4 (8.5)
*Enterobacter cloacae*	3 (6.4)
*Klebsiella aerogenes*	3 (6.4)
*Klebsiella pneumoniae*	3 (6.4)
*Achromobacter* spp.	2 (4.3)
*Citrobacter freundii*	2 (4.3)
*Haemophilus influenzae*	2 (4.3)
*Serratia marcescens*	2 (4.3)
*Klebsiella oxytoca*	1 (2.1)
*Proteus mirabilis*	1 (2.1)
Unknown	11 (23.4)

BAL, bronchoalveolar lavage; CAP, community-acquired pneumonia; CSF, cerebrospinal fluid; HAP, hospital-acquired pneumonia; HSCT, haematopoietic stem cell transplant; IAI, intra-abdominal infection; ICU, intensive care unit; IE, infective endocarditis; SSTI, skin and soft tissue infection; UTI, urinary tract infection; VAP, ventilator-associated pneumonia.

Comorbidities affecting pharmacokinetics in the cohort included any type of renal replacement therapy (27.6%, *n* = 13/47), acute kidney injury (14.9%, *n* = 7/47) or extracorporeal membrane oxygenation (4.2%, *n* = 2/47) Table S1. In spite of the diverse comorbidities, MIPD model fitness metrics were determined to be good or intermediate in >95% (*n* = 45/47) of cases (Table S1). Intermediate fit cases were reviewed by an infectious diseases pharmacist to guide recommendations. The most common type of sample ordered was a peak or trough (96%, *n* = 79/82), consistent with our protocols. Serum concentrations ordered by non-pharmacists (i.e. a protocol deviation) occurred 12.8% (*n* = 6/47) of the time.

### Evaluation metrics

**Figure 1.**
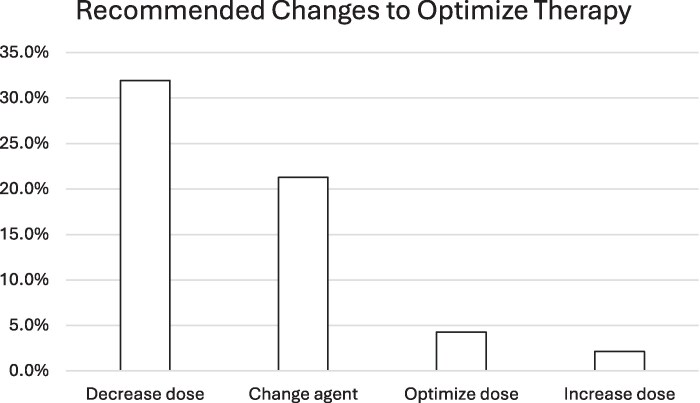
Interventions made to optimize beta-lactam treatment in response to beta-lactam serum concentration data. Optimization of dosing involved changing to an extended (non-standard for cefepime) or continuous infusion (non-standard for all agents).

In response to serum concentration monitoring, clinical pharmacists recommended alterations to the patients’ therapy 60% (*n* = 28/47) of the time (Figure 1). Dose reductions were recommended for 32% (*n* = 15/47) of patients and switching therapeutic agents was recommended 21.3% (*n* = 10/47) of the time. In a single case, treatment was discontinued. In only one case a dose increase was recommended. Doses remained unchanged after MIPD 38.3% (*n* = 18/47) of the time. On the basis of observed serum concentrations and target attainment rates, pharmacokinetic optimization (modification of existing therapy) was possible in 38.3% (*n* = 18/47) of patients (including dose adjustments or changing to extended infusions). Overall, 42.6% (*n* = 20/47) of patients received serum concentration monitoring and MIPD according to protocol. Additional barriers to protocol adherence are summarized in Table 2. Protocol deviations occurring most frequently included incomplete documentation in the EHR, followed by serum concentrations not being evaluated using the MIPD software (i.e. data not entered), levels ordered by non-pharmacists leading to changes made not per protocol, and lack of dose adjustment when indicated. In the initial period of implementation, these types of protocol deviations were anticipated due to a natural learning curve with operationalizing a new workflow. Therapy discontinuation or patient death was considered a protocol deviation due to the inability to assess results of the protocol.

**Table 2.** Patient PK/PD results and impact of serum concentration monitoring on treatment

Endpoint	*n* (%) or median (range)
Achieved 100% *f*T _>_ _MIC_, *n* (%)	43 (91.5)
Median (range) %*f*T _>_ _MIC_	100 (84–100)
Achieved 100% *f*T_>4×MIC_, *n* (%)	29 (61.7)
Median (range) %*f*T_>4×MIC_	100 (5–100)
Median (range) trough	
Cefepime (mg/L), *n* = 36	21.7 (3.7–183.8)
Meropenem (mg/L), *n* = 9^a^	12.9 (1.6–50)
Piperacillin (mg/L), *n* = 1	38.9
Initial trough above safety target	
Cefepime (mg/L), *n* = 36	8 (22.2)
Meropenem (mg/L), *n* = 9^a^	2 (22.2)
Piperacillin (mg/L), *n* = 1	0 (0)
Barriers to protocol	
None	20 (42.6)
Non-adherent with protocol	27 (57.4)
Ordered by pharmacist not entered in software	13 (27.7)
Ordered by non-pharmacist	6 (12.8)
Therapy discontinued	4 (8.5)
User error	2 (4.3)
Laboratory error^a^	1 (2.1)
Patient death	1 (2.1)

^**a**^One patient’s meropenem levels were removed from analysis due to laboratory error.

### Rates of PK/PD attainment and PK parameters

Targets of 100% *f*T_>MIC_ and 100% *f*T_>4×MIC_ were achieved in 91.5% (*n* = 43/47) and 61.7% (*n* = 29/47) of patients, respectively (Table 2), before dose adjustment. The predicted trough concentration was above protocol-defined safety goals in 21% (*n* = 9/46, one patient omitted due to laboratory error) of patients. Figure S2 shows the covariate relationships between drug clearance and covariate values. Among patients not requiring renal replacement, CrCL demonstrated a moderate linear relationship with total drug clearance (*R*^2^ = 0.68) but yielded marginal accuracy and precision (MPE −25%, MAPE 30%). Variability in drug clearance across renal states is visualized in Figure S2a. The relationship between drug clearance and body weight was not strong, as expected for beta-lactam antibiotics in adult patients (Figure S2b).

## Discussion

Beta-lactam dose individualization using serum concentration monitoring identified a need for dose reduction in more than one-third of patients and excessive trough concentrations in more than one in five patients in our cohort. Pharmacokinetic optimization or therapeutic change was possible in 60% of patients underscoring the knowledge gained from obtaining serum concentrations. Our protocol provided guidance to front-line pharmacists on how, when and for whom to perform dose individualization. Although there is not yet consensus guidance on many of these aspects of beta-lactam dose individualization,^15^ our protocol specifically focuses on patients with critical illness and likely PK alterations, those with pathogens with reduced susceptibility or difficult to treat infectious sources and patients at risk for beta-lactam toxicity.

The predominating frequency of dose reductions in our cohort emphasizes the need for vigilance and careful evaluation of neurological alteration in ICU patients. Previous research has suggested that cefepime is associated with neurotoxicity but the exact exposure targets that minimize this risk are not well defined.^16^ We set conservative bounds on trough concentrations in our population, but prospective studies are needed to validate these limits. Concordant with our findings related to dose decreases, Venugopalan *et al.* also observed that cefepime dose decreases were the most common adjustment made in response to cefepime TDM results.^17^ When cefepime is the optimal antibiotic choice for patients, MIPD may be a useful tool in optimizing safety without sacrificing efficacy relating to Gram-negative resistance. In our experience, dose optimization in response to our protocol became another facet of antimicrobial stewardship when alternative therapies had broader spectrum of activity or were less ideal. Our study included BL TDM patients who were receiving cefepime for surgical prophylaxis which may increase heterogeneity of our cohort and limit interpretation of results but were important stewardship cases, nonetheless. In select cases, the risk of cefepime toxicity may be non-trivial due to frequent intra-operative dosing.^18^ Therapy was subsequently adjusted or discontinued as appropriate in response to serum levels exceeding protocolized safety targets, highlighting the opportunity to evaluate appropriateness of continued administration post-operatively.

In our patients, serum beta-lactam concentrations were not predictable based on renal function alone. Cockcroft-Gault calculated creatinine clearance is a population estimate of renal function, which has a coefficient of variation of ∼30% in patients with stable renal function.^19^ We found that CrCL was only modestly predictive of individual drug clearance and had poor accuracy and precision at the patient level. This is consistent with prior research demonstrating variability in beta-lactam exposures in real-world patients. Chang *et al.* used a cefepime PK model in ICU patients to simulate expected PK profiles in cefepime-treated patients at fixed CrCL values and found that the resulting PK exposures ranged 8–30-fold.^20^ Importantly, they found that integration of CrCL provided only a modest improvement in the individual Bayesian predictions compared with their base model, showing that a patient’s CrCL was only slightly useful if their actual PK was also known. Thus, a MIPD approach, which takes into consideration patient-specific PK data, the patient’s covariates (e.g. renal function), and a PK model, is a promising multi-prong approach to help clinicians overcome the high variability in PK exposures seen in real patients.

We anticipated several challenges when we launched our beta-lactam dose individualization programme including institutional barriers (e.g. cost, clinical utility of send out labs, existing practice culture) as well as barriers at the individual clinician level related to perceptions and internalization of the role of beta-lactam TDM.^9,21^ In our evaluation of protocol implementation, we found protocol non-adherence occurring in 57.4% of cases. In the initial months, we identified protocol non-adherence to be consistent with early implementation learning curve (lack of progress note published in the chart, lack of follow-up to level result, inaccurate manual inputs into MIPD software). In response, we offered case-based education sessions to various groups of clinical pharmacists to increase understanding and adherence to protocol steps. In addition, we sought to mitigate institutional barriers by collaborating with the Clinical Laboratory to provide assay results daily during weekdays at midday (in alignment with clinical pharmacists’ workflow) and integrating clinical decision support using a MIPD software for drug level interpretation and providing detailed education to clinical pharmacists. Furthermore, a tip sheet was created and distributed within the first month of protocol implementation in response to observed barriers using new MIPD software.

Nevertheless, we found that a number of patients had levels ordered outside of our protocol and that dose adjustments did not always follow protocol recommendations. Interestingly, all levels ordered by non-pharmacist resulted in changing the therapeutic agent, suggesting a need to carefully steward drug concentrations to ensure proper interpretation. These occurrences were attributed to a lack of education about the protocol to physicians given that the protocol was pharmacist-driven. These findings prompted us to look more deeply at our protocol and to re-engage with stakeholders to maximize the clinical value of our dose individualization programme.^21^ We recognize the need to integrate principles of continuous quality improvement^21^ and continuous education to support internalization of dose individualization.^9^ To this end, we identified pharmacist champions to assist with internalization across our department. These champions met with the infectious disease pharmacists to review individual cases and help reinforce education and refine our protocols over time. We also found that lack of EHR integration of the MIPD software was a barrier, so we worked to facilitate full EHR integration based on our findings. This was implemented after the end of the study period but was anticipated to increase adherence to the protocol process due to eliminating need for manual data entry for each BL TDM level. Over time, protocol deviations due to unfamiliarity with protocol logistics decreased during the study period, largely due to efforts with education coupled with prospective audit and feedback.

Limitations of our study include the single-centre, retrospective observational design. Our evaluation metrics surrogate PK/PD measures and clinical outcomes were not measured. Our study population included mostly cefepime TDM events, which limits generalizability of subsequent changes made to other beta-lactams. However, our study does illustrate real-world challenges likely to be encountered when implementing a beta-lactam dose individualization programme, particularly within the first 6 months of roll-out. Clearly, an adequately powered, well designed, randomized trial of treatment outcomes among patients receiving beta-lactam dose individualization is desirable to help define the efficacy of the intervention and answer the question: what is the impact on outcomes and who is most likely to benefit from dose individualization. However, smaller pragmatic descriptive studies such as ours highlight important challenges and can suggest potential solutions when implementing beta-lactam dose individualization in the real world. For example, finding that many serum levels were ordered outside of protocol provides opportunity for education and provider engagement. Most of the serum concentration monitoring events in our cohort were for cefepime, which limits generalizability to other drugs. Our protocol relied on in-house assays and a pharmacist-driven stewardship infrastructure which limits generalizability to institutions without such resources. Additional studies are needed to validate the efficacy and safety targets used in our protocol.

### Conclusion

Most patients achieved 100% *f*T_>1×MIC_ based on initial serum concentration monitoring evaluated using MIPD software. However, more than two-thirds of patients in our cohort required per-protocol modifications to therapy in response to serum concentration monitoring. Pharmacokinetic optimization was possible in 60% of patients and trough concentrations exceeded safety targets in >20% of patients based on our local protocols. Protocol deviations were common suggesting that ongoing effort is needed to sustain beta-lactam dose individualization programmes to ensure optimal care of patients.

## Supplementary Material

dlag047_Supplementary_Data

## Supplementary data

Figures S1 and S2 and Tables S1 and S2 and Supplementary Methods are available as Supplementary data at *JAC-AMR* Online.
