# Supplementary material for: Individualizing beta-lactam dosing in real-world patients: lessons from a pharmacist-led programme implementation and evaluation
Source: JAC Antimicrob Resist. 2026 Apr 8;8(2):dlag047. doi: 10.1093/jacamr/dlag047 (PMC13065389; doi:10.1093/jacamr/dlag047)
Supplement: dlag047_Supplementary_Data [file dlag047_supplementary_data.docx]

**Supplemental Appendix**

Supplemental Methods: Serum drug assay

All four analytical standards (cefepime, meropenem, piperacillin, tazobactam) and their internal standards (cefepime-^2^H_8_, meropenem-^2^H_6_, piperacillin-^2^H_5_, tazobactam-^15^N_3_) were purchased from commercial suppliers. Solutions of analytical standards were made in dimethylsulfoxide at 50 mg/mL each, and solutions of internal standards at 1 mg/mL each; all solutions were stored at -80^o^C. The analytes were quantified in serum by liquid chromatography coupled to tandem mass spectrometry with Waters Acquity UPLC® components (binary pump, sample manager, column manager) and a Waters Xevo TQ triple quadrupole detector (electrospray ionization, positive-ion mode). Two transitions were monitored for each analyte, and data were gathered in MRM mode. Chromatography was effected on a Waters Acquity UPLC® BEH C18 column (1.7μ, 2.1×100mm), held at 30^o^C in a mobile phase comprising water + 0.1% formic acid (solvent **A**) and acetonitrile + 0.1% formic acid (solvent **B**), flowing at 0.3 mL/min. Total run time was 5.0 minutes. There were three step gradients in the pump method: 98% **A** (0→0.3 min), 60% **A** (0.3→2.1 min), 98% **A** (2.1→5.0 min). Samples were extracted by mixing 50 μL of serum and 150 μL of acetonitrile containing the four internal standards, each at 1.0 μg/mL. Extracts then stood at room temperature for 10 minutes and were centrifuged at 16,000*g* for five minutes. Of the supernatant, 100 μL was removed and mixed with 300 μL of water; this extract was injected for analysis. All calibrators, controls, and patient specimens were stored at -80^o^C. Each calibration curve comprised five points and was weighted by 1/*x*.

**Figure S1.** Workflow of a Pharmacist-Driven Beta-lactam Dose Individualization Program

**Abbreviations:** BAL: bronchoalveolar lavage; CRRT: continuous renal replacement therapy; ECMO: extracorporeal membrane oxygenation; EHR: electronic health record; MIC: minimum inhibitory concentration; MIPD: model-informed precision dosing; PK/PD: pharmacokinetics/pharmacodynamics; TDM: therapeutic drug monitoring.

**Table S1.** Characteristics of serum concentrations sampling events

| **Characteristics** | **n (%)** |
| --- | --- |
| Level by antibiotic, N=82 levels |  |
| Cefepime | 58 (70.7) |
| Meropenem | 21 (25.6) |
| Piperacillin | 3 (3.7) |
| Level type, N=82 levels |  |
| Trough | 43 (52.4) |
| Peak | 36 (43.9) |
| Random | 3 (3.7) |
| Model Fit, N=47 patients |  |
| Good | 35 (74.5) |
| Intermediate | 10 (21.3) |
| Poor | 2 (4.3) |
| Model used, N=47 patients |  |
| Adult / Intensive care (Jonckheere, AAC 2019) [1] | 25 (53.2) |
| Li C. et al. J Clin Pharm 2006 [2] | 8 (17) |
| Adult / Sepsis (Delattre IK, Clin Biochem 2012) [3] | 7 (14.9) |
| Adult / Intensive care (An G, JAC 2023) [4] | 6 (12.8) |
| Andersen MG, AAC 2018 [5] | 1 (2.1) |
| Ordering provider, N=47 patients |  |
| Pharmacist | 41 (87.2) |
| Non-pharmacist | 6 (12.8) |
| Treatment Flags, N=47 patients |  |
| AKI | 7 (14.9) |
| CRRT | 7 (14.9) |
| iHD | 5 (10.6) |
| Peritoneal dialysis | 1 (2.1) |
| ECMO | 2 (4.2) |
| None | 25 (53.2) |

**Abbreviations:** AKI: acute kidney injury; CRRT: continuous renal replacement therapy; ECMO: extracorporeal membrane oxygenation; iHD: intermittent hemodialysis.

**Table S2.** Institutional Empiric Cefepime Renal Dosing Protocol During Study Period

| Medication | Indications & Comments | Usual Dose  CrCl >50 mL/min | CrCl 31-50 mL/min | CrCl 10-30 mL/min | CrCl < 10 mL/min | Anephric/HD* |
| --- | --- | --- | --- | --- | --- | --- |
| Cefepime (infused over 0.5 hr) | Severe dosing (e.g. neutropenic fever, CNS, sepsis, osteomyelitis) | 2 g every 8h | 2 g every12h | 2 g every 24h | 2 g LD, then 1 g every 24h | 1 g every 24h  or 2 g 3x weekly post-HD * |
|  | Cystitis or less severe infections in patients <55kg or elderly (≥80 years) | 1 g every 8h | 1 g every 12h | 1 g every 24h | 1 g every 24h | 1 g every 24h or  2 g 3x weekly post-HD * |

**Abbreviations:**  CNS, central nervous system; CrCL, estimated creatinine clearance (Cockcroft-Gault); LD, loading dose; HD, intermittent hemodialysis. **Footnote:** *When dosing antibiotics for patients on intermittent hemodialysis (HD), recommend scheduling dosing to be giving after HD sessions (e.g., schedule Q 24h at 17:00). Recommended order comment wording: “Give at the scheduled frequency (including non-HD days if scheduled. On HD days, give after HD”.

**Figure S2.** Relationship between estimates CrCL and Bayesian predicted drug clearance values

**Abbreviations:** CrCL: Cockcroft-Gault creatinine clearance; RRT: renal replacement therapy.

**References:**

1 Jonckheere S, De Neve N, Verbeke J *et al*. Target-Controlled Infusion of Cefepime in Critically Ill Patients. *Antimicrob Agents Chemother* 2019; **64**.

2 Li C, Kuti JL, Nightingale CH *et al*. Population pharmacokinetic analysis and dosing regimen optimization of meropenem in adult patients. *J Clin Pharmacol* 2006; **46**: 1171-8.

3 Delattre IK, Musuamba FT, Jacqmin P *et al*. Population pharmacokinetics of four beta-lactams in critically ill septic patients comedicated with amikacin. *Clin Biochem* 2012; **45**: 780-6.

4 An G, Creech CB, Wu N *et al*. Population pharmacokinetics and target attainment analyses to identify a rational empirical dosing strategy for cefepime in critically ill patients. *J Antimicrob Chemother* 2023; **78**: 1460-70.

5 Andersen MG, Thorsted A, Storgaard M *et al*. Population Pharmacokinetics of Piperacillin in Sepsis Patients: Should Alternative Dosing Strategies Be Considered? *Antimicrob Agents Chemother* 2018; **62**.
